# Exploring cognitive and brain oxygenation changes over a 1-year period in physically active individuals with mild cognitive impairment: a longitudinal fNIRS pilot study

**DOI:** 10.1186/s12877-022-03306-x

**Published:** 2022-08-08

**Authors:** Deborah Talamonti, Christine Gagnon, Thomas Vincent, Anil Nigam, Frederic Lesage, Louis Bherer, Sarah Fraser

**Affiliations:** 1 Research center and EPIC Center, Montreal Heart Institute, Montreal, QC Canada; 2 Department of Medicine, University of Montreal, Montreal, QC Canada; 3 École Polytechnique de Montréal, Montreal, QC Canada; 4 Centre de recherche, Institute universitaire de gériatrie de Montréal, Montreal, QC Canada; 5 Interdisciplinary School of Health Sciences, University of Ottawa, Ottawa, ON Canada

**Keywords:** Dual-task, Walking, NIRS, Hemodynamics, Cognitive impairment, Older adults, Physical activity

## Abstract

**Background:**

Aging is associated with an increased likelihood of developing dementia, but a growing body of evidence suggests that certain modifiable risk factors may help prevent or delay dementia onset. Among these, physical activity (PA) has been linked to better cognitive performance and brain functions in healthy older adults and may contribute to preventing dementia. The current pilot study investigated changes in behavioral and brain activation patterns over a 1-year period in individuals with mild cognitive impairment (MCI) and healthy controls taking part in regular PA.

**Methods:**

Frontal cortical response during a dual-task walking paradigm was investigated at baseline, at 6 months (T6), and at 12 months (T12) by means of a portable functional Near-Infrared Spectroscopy (fNIRS) system. The dual-task paradigm included a single cognitive task (2-back), a single motor task (walking), and a dual-task condition (2-back whilst walking).

**Results:**

Both groups showed progressive improvement in cognitive performance at follow-up visits compared to baseline. Gait speed remained stable throughout the duration of the study in the control group and increased at T6 for those with MCI. A significant decrease in cortical activity was observed in both groups during the cognitive component of the dual-task at follow-up visits compared to baseline, with MCI individuals showing the greatest improvement.

**Conclusions:**

The observations of this pilot study suggest that taking part in regular PA may be especially beneficial for both cognitive performance and brain functions in older adulthood and, especially, in individuals with MCI. Our findings may serve as preliminary evidence for the use of PA as a potential intervention to prevent cognitive decline in individuals at greater risk of dementia.

**Supplementary Information:**

The online version contains supplementary material available at 10.1186/s12877-022-03306-x.

## Background

Due to the unprecedented increase in life expectancy, it is estimated that by 2050 there will be 2 billion individuals, nearly one-fifth of the world’s population, aged ≥60 years [1]. As a consequence, the risk of age-related chronic conditions and diseases will increase critically. One condition that emerges in older adulthood and that has significant implications for health and economical institutions is cognitive decline. Although at certain levels cognitive decline is considered a normal consequence of the aging process, abnormal cognitive decline causes objective or subjective impairments that may interfere with daily functioning and be a precursor of future dementia [2]. To date, no definitive treatment exists for dementia. Therefore, early prevention, as well as early diagnosis, are currently of great clinical ﻿importance [3].

An increasing body of research has suggested that lifestyle habits, such as healthy eating, being cognitively active, and regular physical activity (PA) may significantly decrease the number of future dementia cases [3﻿], improve neural resources (i.e., cognitive reserve) [4] and quality of life of older individuals in the long term [5]. In healthy older adults, regular PA has been associated with more efficient cognitive functions [6], especially executive functions [7], and with a decreased risk of developing Alzheimer’s disease (AD), the most common form of dementia [8]. Among the underlying mechanisms, it has been suggested that regular PA promotes cerebrovascular integrity by improving cardiovascular health [9] and stimulating neurogenesis in the brain [10], as well as by providing optimal blood flow, nutrients and oxygenation to the brain [11]. Brain imaging studies have reported PA benefits specific to frontal and temporal areas (e.g., greater grey and white matter, larger cortical and hippocampal volumes), which play a crucial role in the pathogenesis of AD [12]﻿. Given the vast benefits of PA, the latest international guidelines on healthy aging strongly recommend regular PA in adults with normal cognitive functioning to promote healthy aging [13﻿]. The effects of PA in individuals with cognitive impairment or dementia are still under investigation [﻿^14^–^16^]. Recent meta-analyses have suggested a potential protective effect of PA in the progression to mild cognitive impairment (MCI), the prodromal stage of dementia, and to AD [17, 18]. For instance, Lautenschlager et al. [19] showed that 6 months of participation in regular PA resulted in an improvement of general cognitive function in individuals at risk of AD, whereas a deterioration in cognition was observed in those who did not participate in regular PA. Similarly, Etgen et al. [20] observed that 2 years of high PA was related to stable levels of cognitive functioning in older adults with MCI, whereas individuals engaged in moderate or no PA showed a significant decrease in cognitive performances. These findings suggest that engaging in regular PA may be especially beneficial in this population to reverse or slow the trajectory of abnormal cognitive declines [21]. Although investigations on the relationship between PA and progression to MCI and dementia are substantial [22], few studies have to date investigated the effects of regular PA in MCI overtime. To our knowledge, no study has so far investigated longitudinal changes in both brain functions and cognitive performance in MCI individuals taking part in regular PA, albeit such research is crucial to understand the role that PA may play in dementia prevention.

Declines in cognitive functions or walking speed have been identified separately as early markers of cognitive decline and dementia [23–25], with decline being detectable up to a decade before the onset of dementia [26, 27]. Recently, concomitant rather than separate decline in cognitive performance and walking speed has been shown to increase risk of dementia by three times compared to decline in only one domain [25]. In this context, dual-task experimental designs are especially suited to explore the behavioral and task-related brain changes due to PA. Dual-task walking paradigms are based on the ability to divide attention between cognitive and motor activities, which becomes especially challenging in older adulthood [28]. Typically, such paradigms consist of two single tasks, one cognitive and one motor, versus a condition wherein cognitive and motor tasks are performed simultaneously. Dual-task walking paradigms have been demonstrated to be sensitive to age-related decline [24, 28] and to accurately measure cognitive and physical improvements induced by PA and exercise [29]. Brain imaging studies using fNIRS technology have reported that prefrontal cortical oxygenation typically increases proportionally to the cognitive load (single-task vs. dual-task) and is related to cognitive and motor performance [30, 31], although alternative patterns may be observed in older adults [24]. For instance, preliminary findings in individuals with cognitive impairment (MCI) showed greater hemodynamic response (i.e., increased oxygenated hemoglobin levels) during dual-task walking compared to single walking [32]. However, such overactivation was related to slower, rather than faster, walking speed during the dual-task condition compared to single walking. Recently, this pattern was found to be attenuated when participants with MCI were compared to healthy controls [33]. Although a decline in motor abilities is a natural consequence of aging, significant decline, especially in walking speed, has been linked to future cognitive decline and dementia [34].

Regular PA has been suggested to alleviate age-related motor decline in healthy older adults, and to potentially protect against the risk of dementia, thus emphasizing the holistic beneficial effect of PA in older adulthood [35]. A recent meta-analysis also showed that physical exercise had a positive effect on cognition (i.e., general cognitive functioning, executive functions, attention and delayed memory) in older individuals with MCI [36]. In the present pilot study, we investigated changes in behavioral and brain activation patterns over a 1-year period in individuals with MCI and healthy controls taking part in regular PA. During the dual-task walking paradigm, behavioral performance changes were captured, as well as, related neural changes in frontal activity measured with functional Near-Infrared Spectroscopy (fNIRS). We expected that all participants would improve their behavioral performances over the 12-month period of participation in regular physical activity at the dual-task walking paradigm and would show more efficient task-related brain oxygenation (i.e., reduced rather than increased cortical activation), with MCI showing the most improvement.

## Methods

### Participants

Participants were part of a longitudinal project investigating the link between participation in regular physical activity, cardiovascular risk factors and cognitive decline. All participants were members of the Centre de médecine préventive et d’activité physique (EPIC Center) or the Montreal Heart Institute, one of the largest cardiovascular prevention centers in Canada and in North America. Participants for this study were selected if aged 50 or above, right-handed, completed the 6- and 12-month follow-up visits, had no history of neurological, as well as psychiatric conditions. From the pool of sixty-eight participants, we analyzed data from thirty-two volunteers who fulfilled the inclusion criteria for the present study. The MCI label was given to participants based on NIA-AA and Petersen’s criteria [37, 38], including impairment in one or more cognitive domains (− 1 to − 1.5 SD below the average), as reflected by a score lower than 26 on the MoCA [39] and by z-scores on the immediate and delayed memory performance based on the Rey Auditory Verbal Learning Task (RAVLT [40﻿];). Although functional abilities were not assessed in the present study, all participants were community dwelling and independent older adults that were able to provide consent and take part in the study. As part of the inclusion criteria for the study, participants did not have neurological or psychiatric conditions, including dementia.

### Procedure

**Fig. 1:**
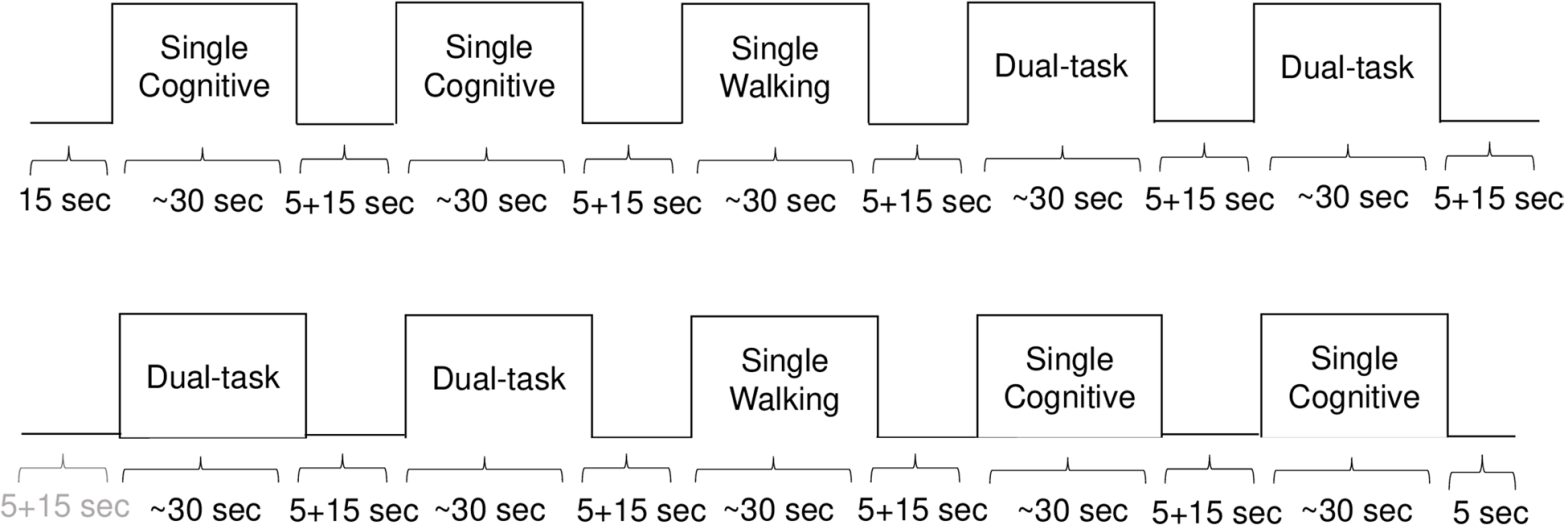
Description of the experimental design

Each participant completed an entry assessment that included: informed consent, clinical and physical measurements, psychological status, neuropsychological, and fNIRS assessments. The assessments were repeated at 6 months and at 12 months, and included commonly used clinical tests and questionnaires, including measures used for this study: the MoCA [39], the AVLT [40], the Trail Making Test (TMT [41﻿];), the Geriatric Depression Scale (GDS [42﻿];), the Physical Activity Scale for the Elderly (PASE [43﻿];), and the dual-task walking paradigm. The dual-task paradigm consisted of three experimental conditions: single cognitive task (SC), single walking task (SW), and dual-task (DT), combining both single tasks. The single cognitive task (SC) required participants to perform the n-back task, which is a commonly used working memory task in which participants listen to series of numbers and then are asked to name the number presented “n” positions back. For example, in the 2-back version used in this study, the participant hears 2-3-5-1-8; when the participant hears 5, he has to say 2; 1 say 3, etc. To ensure adequate comprehension of the task and better than chance ability on the task before testing, participants had to achieve 60% or above on the single cognitive condition before proceeding with testing. Participants were presented one digit every 1.5-2 seconds, with an interstimulus interval of 1.5 seconds in which participants could respond. For each trial, participants were presented a total of 10 digits, if they were able to correctly respond to all 10 digits, they received 100% accuracy score. During the single walking task (SW) participants were asked to freely walk back and forth on a 10-m track for 30-second, whilst wearing a head-fixed accelerometer. During the dual-task (DT), participants were asked to complete the auditory 2-back task whilst walking. Each experimental condition was administrated in blocks of 30 seconds, for a total of 10 blocks, with periods of rest lasting 5 seconds before each block and 15 seconds after each block. The complete sequence of experimental blocks was as follows: SC – SC – SW – DT – DT – DT – DT – SW – SC – SC (Fig. 1). Gait speed (m/s) and accuracy on the cognitive task (percentage of correct responses) were measured at each trial and averaged over the trials for each condition. During the dual-task conditions, participants’ hemodynamic response was recorded by means of a portable fNIRS system (details on the fNIRS in the following section).

Following the entry assessment, participants were recommended to take part in any exercise training or physical activity at least twice per week at the ÉPIC Center or elsewhere. Given the observational nature of this study, PA was not directly monitored throughout the study. However, weekly frequency, duration, and intensity of PA were obtained through self-reported questionnaires. The Borg scale of perceived exertion was used by participants to record the intensity of physical activity [44]. The PASE was used to identify the level of physical activity (hours/week) of participants at 6-month and at 12-month follow-ups.

### fNIRS recording and signal extraction

**Fig. 2:**
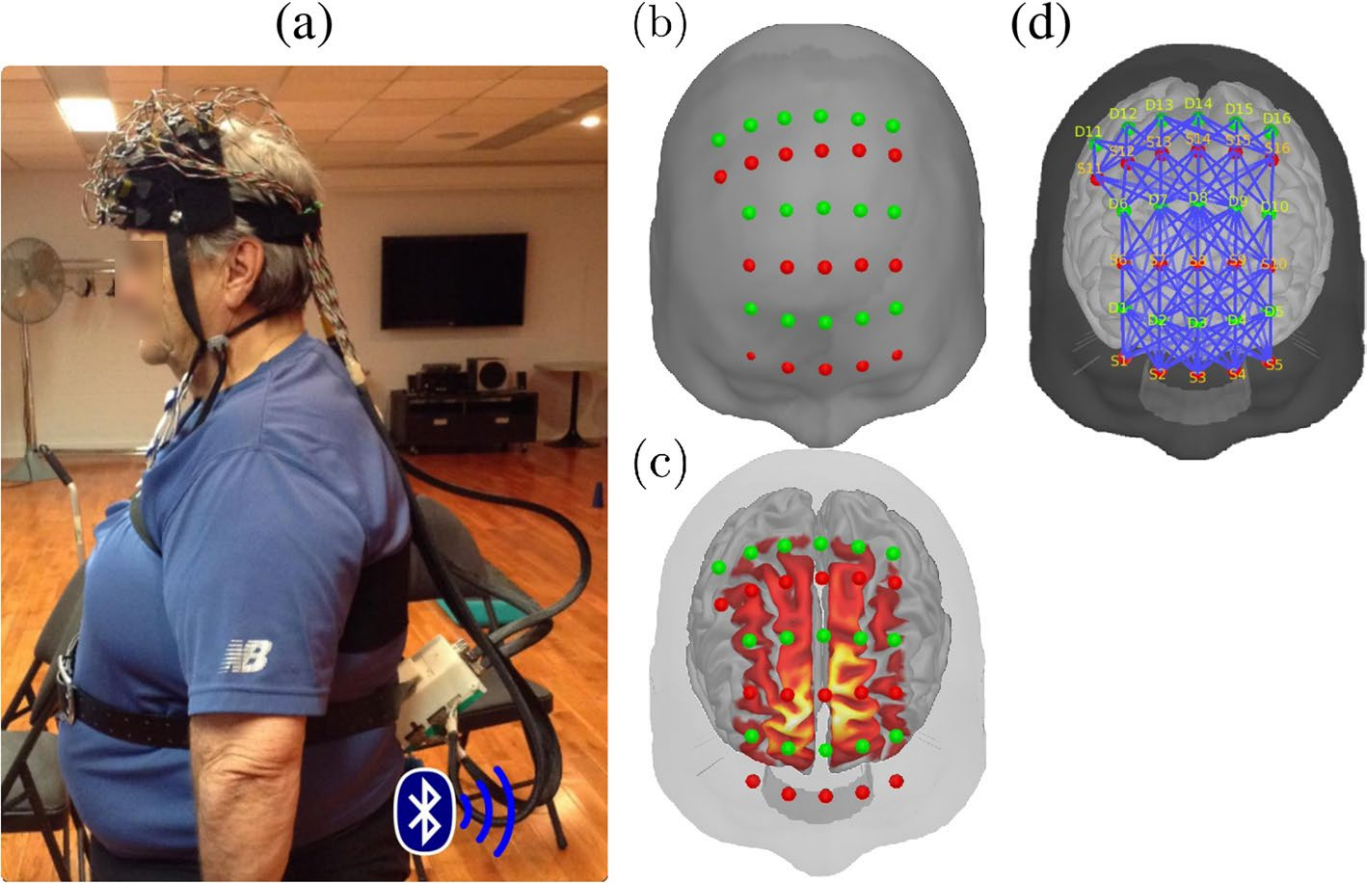
Functional Near-Infrared Spectroscopy (fNIRS) system, optodes configuration, and experimental setup

Hemodynamic variations were acquired during the dual-task paradigm, described in the previous section, using a portable fNIRS in-house prototype [45] (Fig. 2). Sample frequency was set at 20 Hz, and changes in light attenuation were measured at two wavelengths (735 and 850 nm). Sixteen detectors and sixteen sources were placed symmetrically over the prefrontal cortex, with an average source-detector separation of 2.8 cm, using a stretchable band. The montage was aligned so that the middle source of the bottom row was placed on Fpz, according to the international 10-20 EEG system (Electrode Position Nomenclature Committee, 1994). This configuration allowed coverage of the frontal and part of the motor cortices, by pairing each source to its 8 closest detectors for a total of 128 pairs and 256 channels (2 wavelengths per pair). When needed, hair was removed from the optical fibers to maximum light transmission and prevent exaggeration of motion artefacts. An acquisition box attached to the participant’s belt was wired to the optodes and transmitted data to the acquisition PC via Bluetooth. Signal quality was verified with heart beats clearly visible before and during the measurement.

### fNIRS signal processing

**Fig. 3:**
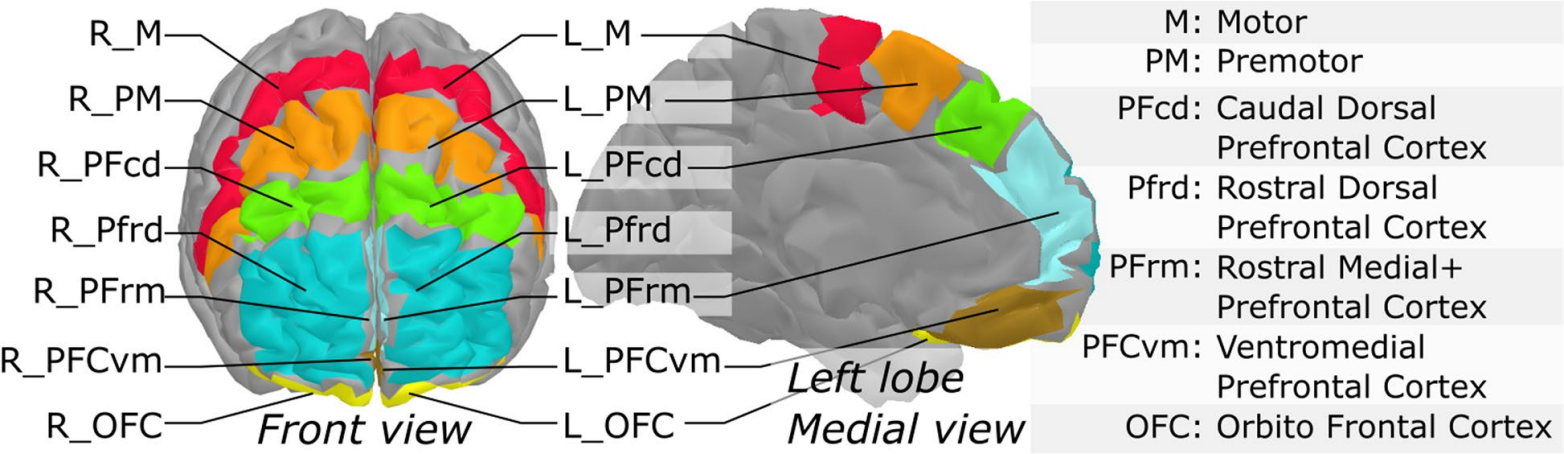
Segmentation of the prefrontal cortex based on MarsAtlas applied to the Colin27 atlas. These regions were used to produce region-averages of NIRS task-related effect sizes. Orientation: the left side of the figure is Participant’s right

fNIRS signals were processed under Matlab using the brainstorm toolbox [46] and the nirstorm plugin [47]. The first step involved the removal of bad channels. Channels were excluded when signals showed saturation periods, large changes or a high amount of noise without the presence of heart beats. Around 60% of optode pairs were removed, mostly long-distance ones (more than 4 cm). Given the nature of the experimental design, which included conditions especially prone to motion artifacts (e.g., walking conditions), motion artefacts were manually tagged by identifying short-lived periods (max 2 sec) of abrupt changes spread across several channels. Based on this tagging, motion correction was applied using a spline-based interpolation method [48]. A bandpass filter between 0.01 and 0.1 Hz was applied to only keep the evoked hemodynamic band. Head signals were then projected on the cortical surface of the Colin27 template [49] using the Minimum Norm Estimate algorithm [50]. Cortical signals were then converted to variations of [HbO] and [HbR] by using the hemoglobin extension coefficients. A first-level GLM with a pre-colored noise model [51] was applied to the cortical time-series of each subject to obtain within-subject maps of [HbO] and [HbR] changes evoked by the working memory task. Since the NIRS spatial resolution is relatively low, mesh-based cortical mappings contain redundant information. To get a more parsimonious representation of NIRS mappings, regional averages were computed using a coarse version of the MarsAtlas cortical parcellation [52]. This segmentation consisted of a set of 14 regions of interest (ROIs; 7 per hemisphere), as depicted in Fig. 3 with the list of region labels. Lastly, to keep only the areas that were potentially engaged in the experimental paradigm, task-specific functional masks were computed from a group-level analysis. This allowed to optimize sensitivity rather than specificity and to selectively catch regions that were potentially involved in the tasks and may have interacted with the behavioral variables. To do so, a non-conservative strategy using a second-level GLM with a mixed-effect noise model [51] was applied to produce binary maps from t-stats thresholded at *p* < .05 (uncorrected). The second-level GLM analysis provides a global functional mask of regions that are potentially involved in the task. For each experimental condition, this allowed to filter out the regions that elicited no activity at the group-level. The regional approach, with five regions considered, allowed to control for potential multiple comparison issues. Within-region and within-subject maps were computed as the effect size (estimated GLM effect magnitude divided by its standard deviation), to take into account temporal fluctuations that may vary across regions and participant.

### Statistical analysis

First, independent-samples t-tests were performed to investigate between-group differences on clinical characteristics. Second, a series of mixed ANCOVAs were performed to explore intra- and inter-individual differences and the effect of time on behavioral performance and brain hemodynamics, whilst controlling for sex and education, due to differences between the groups on these variables. Specifically, the ANCOVAs investigated: n-back performance (accuracy) (Group: controls, MCI × Time: T0, T6, T12 × Condition: SC, DT), gait speed (m/s) (Group: controls, MCI × Time: T0, T6, T12 × Condition: SW, DT), HbO and HbR responses in each activated ROI respectively during SC, SW and DT (Group: controls, MCI × Time: T0, T6, T12). Post-hoc analyses were performed using Bonferroni correction. Finally, correlation analyses were performed to investigate the association between behavioral performance and cerebral oxygenation during dual-task walking, in each group, condition and at each time point.

## Results

Baseline demographic information and characteristics of the physical activity are reported in Tables 1 and 2. Data are mean ± standard deviation, unless otherwise stated. The two groups showed no differences regarding presence of cardiovascular risk factors or regarding their participation in physical activity in terms of weekly frequency and duration. Of note, group differences in training intensity were reported at T6, where participants with MCI reported significantly more intense PA compared to healthy controls [*t* (30) = − 2.116, *p* = .044]. The two groups showed differences in level of education [*t* (30) = − 2.832, *p* = .011] and sex [*t* (30) = 2.319, *p* = .027]. Specifically, the group with MCI reported fewer years of education and had fewer female participants than the control group. These differences were taken into consideration for the main statistical analyses, wherein sex and education were included as covariates.

**Table 1:** Demographic characteristics

Variables	Controls	MCI
Sample size n (%)	24 (75%)	8 (25%)
Female (%)	**14 (58.3%)**	**1 (12.5%)**
Age (years)	67.91 ± 5.42	71.50 ± 4.11
Education	**16.96 ± 3.29**	**13.25 ± 4.50**
Presence of CVRF n (%)		
Diabetes	5 (20.9%)	3 (37.5%)
Smoking	3 (12.5%)	1 (12.5%)
Hypertension	10 (41.7%)	5 (62.5%)
Dyslipidemia	12 (50%)	6 (75%)
Family history	12 (50%)	4 (50%)
Obesity	6 (25%)	3 (37.5%)

*Note*. Results are mean ± SD. *CVRF* cardio-vascular risk factors. Highlighted difference between groups *p* < .05

**Table 2:** Clinical information and characteristics of physical activity

	T0		T6		T12	
Variables	Controls	MCI	Controls	MCI	Controls	MCI
MoCA	**27.79 ± 1.32**	**24.12 ± 0.83**	**27.00 ± 1.59**	**25.12 ± 2.36**	**27.33 ± 1.52**	**25.12 ± 1.73**
RAVLT immediate recall	**47.37 ± 8.73**	**37.25 ± 8.36**	**46.29 ± 8.07**	**37.25 ± 9.22**	**49.12 ± 8.99**	**35.50 ± 9.71**
RAVLT delayed recall	**10.58 ± 2.84**	**6.37 ± 2.77**	**10.33 ± 3.02**	**5.87 ± 3.31**	**10.62 ± 2.57**	**6.00 ± 4.47**
TMT-A	34.00 ± 10.81	33.00 ± 4.20	37.79 ± 11.50	38.71 ± 9.07	37.92 ± 15.46	35.37 ± 6.99
TMT-B	78.92 ± 25.21	92.75 ± 30.35	81.04 ± 27.01	80.50 ± 18.85	79.42 ± 37.03	88.25 ± 20.27
GDS	4.25 ± 3.08	3.37 ± 4.60	3.96 ± 4.14	3.62 ± 5.88	3.50 ± 4.43	4.00 ± 7.52
PASE	137.10 ± 67.95	87.35 ± 39.43	106.19 ± 67.41	87.61 ± 31.19	118.36 ± 59.89	99.62 ± 62.27
Characteristics of the physical activity						
Frequency (n° visits/week)	–	–	1.96 ± .93	2.03 ± .76	1.64 ± .96	1.99 ± 1.26
Duration (min/week)	–	–	250.81 ± 197.63	276.20 ± 145.4	195.81 ± 102.39	290.27 ± 192.45
Intensity (Borg’s scale)	–	–	**4.16 ± 1.90**	**5.27 ± 1.56**	3.87 ± 2.29	5.26 ± 2.04

*Note.* Results are mean ± SD

*T0* Baseline, *T6* 6-month follow-up, *T12*, 12-month follow-up, *MoCA* Montreal Cognitive Assessment, *RAVLT* Rey Auditory Verbal Learning Test, *TMT A & B* Trail Making Test A & B, *GDS* Geriatric Depression Scale, *PASE* Physical Activity Scale for the Elderly. Highlighted difference between groups *p* < .0

General cognitive functioning was significantly lower in the MCI group than in controls at baseline [t (30) = 7.347, *p* < .001], at T6 [t (30) = 2.556, *p* = .016], and at T12 [t (30) = 3.439, *p* = .002]. Immediate and delayed memory performance was lower in the MCI group than in controls at baseline [RAVLT immediate recall: t (30) = 2.869, *p* = .007; RAVLT delayed recall: t (30) = 3.467, *p* = .001], at T6 [RAVLT immediate recall: t (30) = 2.652, *p* = .013; RAVLT delayed recall: t (30) = 3.536, p = .001], and at T12 [RAVLT immediate recall: t (30) = 3.641, p = .001; RAVLT delayed recall: t (30) = 3.634, *p* = .022]. No group differences were reported for the TMT at the three time points.

### Behavioral results

**Fig. 4:**
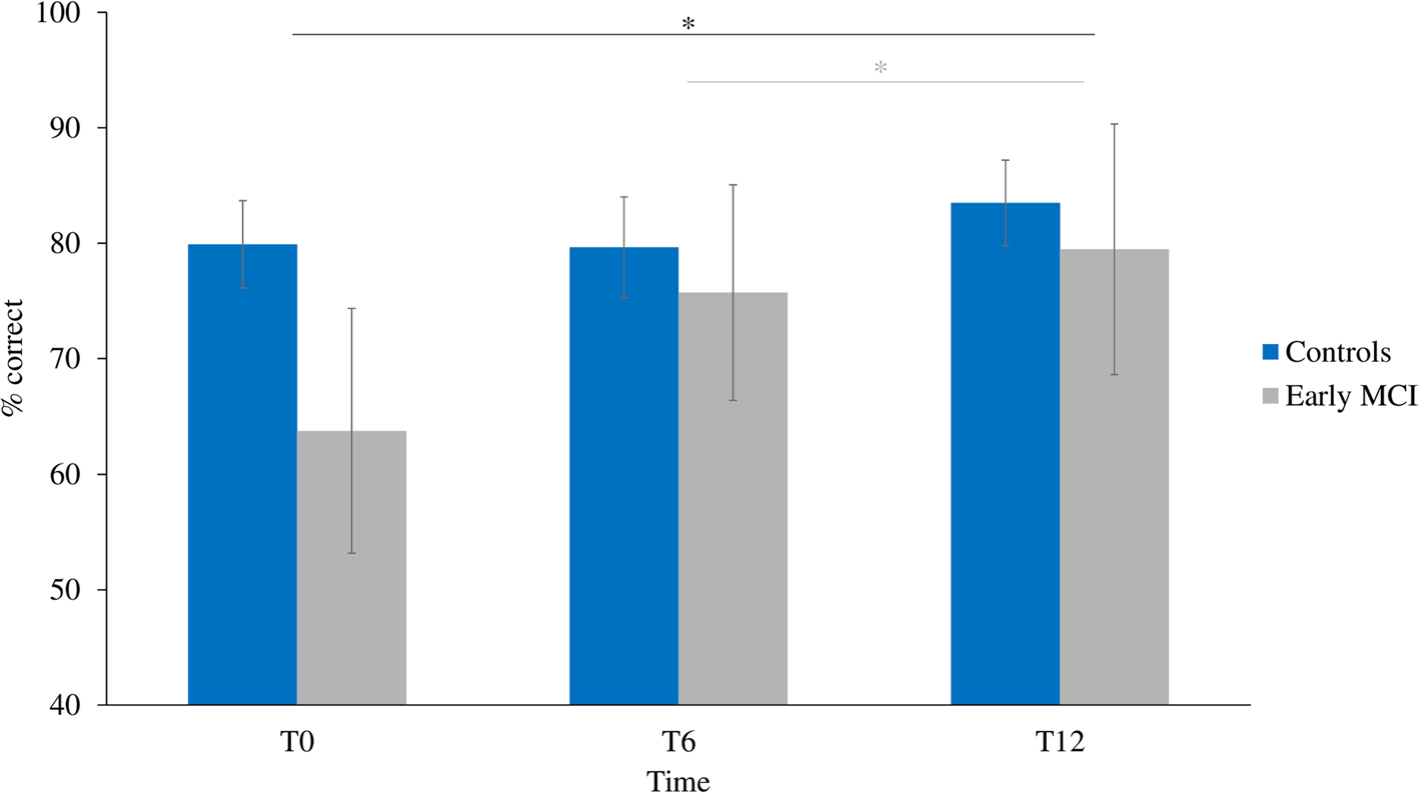
Overall cognitive performance at each time point. Bars indicate standard error. * = *p* < .05

The ANCOVA performed on n-back task performance (% correct responses on the n-back during single and dual-task conditions) revealed no significant main effects of group or condition and no significant interactions. A significant main effect of time was found [F(2, 56) = 3.915, *p* = .038, η2p = .123], in which cognitive performance was greater at T12 than at T0 (*p* = .031) and tended to significance at T6 (*p* = .051) in all participants, regardless of the experimental condition, as shown in Fig. 4.

**Fig. 5:**
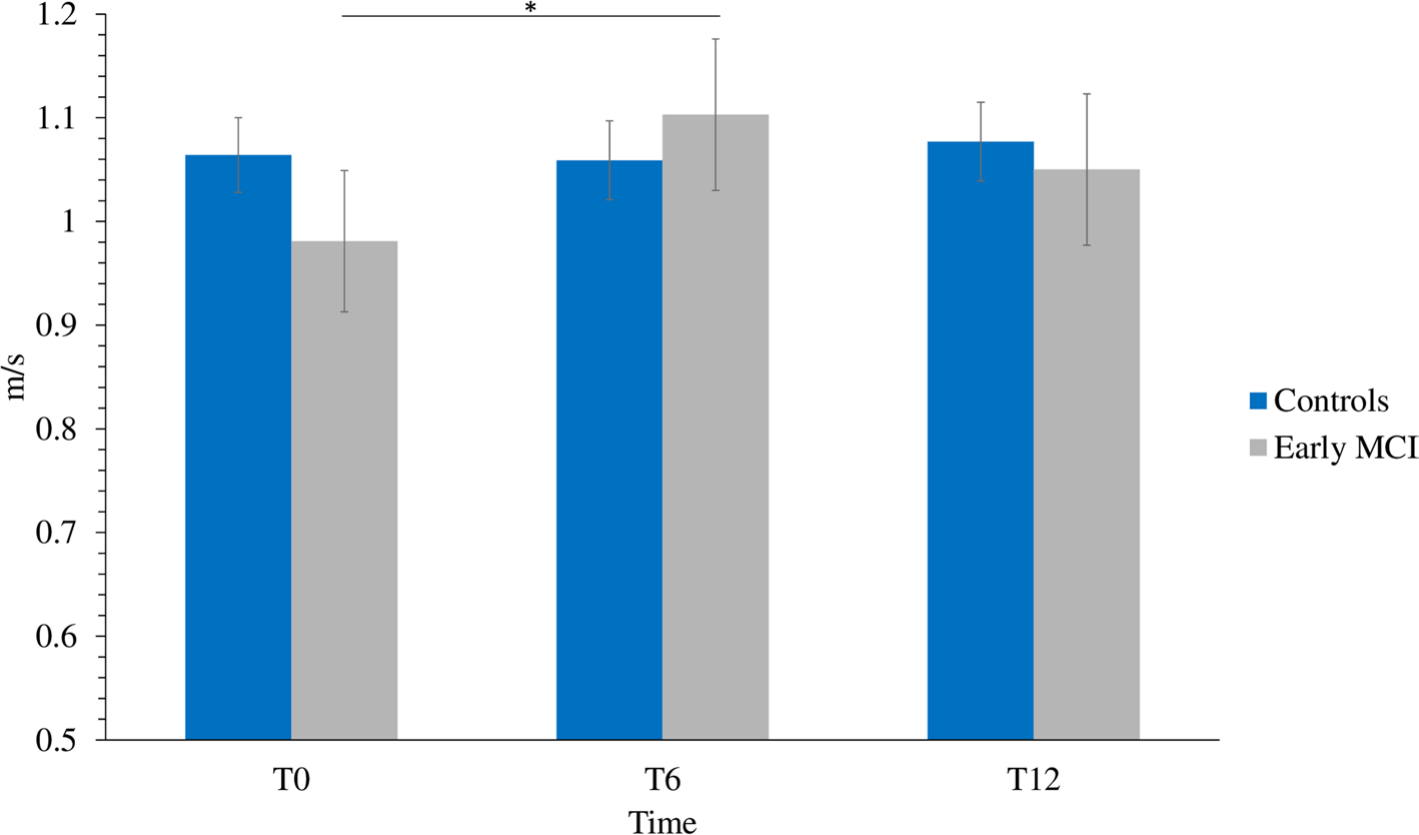
Overall gait speed at baseline (T0), 6-month (T6) and 12-month (T12) follow-up visits. Bars indicate standard error. * = *p* < .05

The ANCOVA investigating gait speed (m/s) revealed no main effects of group, condition or time. A significant time by group interaction was found [F(2, 56) = 3.232, *p* = .047, η2p = .103] in which the group with MCI walked faster at T6 than at T0 regardless of the experimental condition (SC or DT) (*p* = .037) (Fig. 5). There were not statistically significant differences between T12 and T0 and no other significant interactions. Walking speed for healthy controls remained stable at the different time points (Table 2).

### fNIRS results

**Fig. 6:**
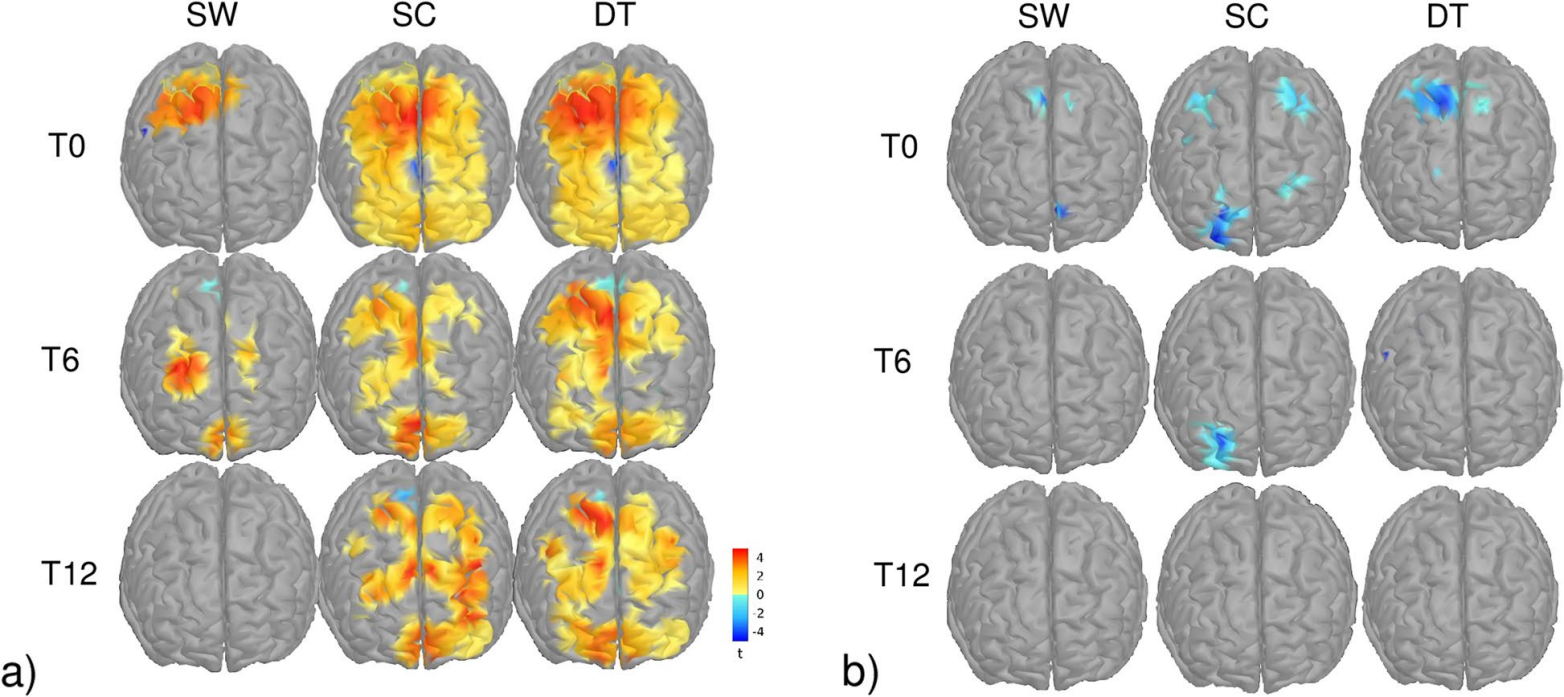
Second-level cortical mappings of HbO (a) and HbR (b) changes evoked by Single Walking (SW), Single Cognitive (SC), and Dual Task (DT) conditions computed from all participants at baseline (T0), 6-month follow-up (T6), and 12-month follow-up (T12), thresholded at *p* < .05

fNIRS results grouping participants from both groups showed bilateral activation of frontal areas that was proportionate to the complexity of the tasks (Fig. 6). At baseline, the hemodynamic response was lower during SW, limited mainly to premotor and motor areas (PM, M, PFcd), and proportionally greater during SC and DT, where prefrontal regions were especially recruited (PFrm, PFcd, PFrd, PM, M). The hemodynamic response for HbR values was limited to the motor and prefrontal regions during SC and DT (Pfcd, PFrd, PM, M) and to premotor regions during SW.

Mean and standard deviations of HbO responses at each time point and for each group are reported in Table 1S of the supplementary materials. These values were used for the following analyses. First, the 2 × 2 ANCOVAs investigating group-related and time-related cerebral oxygenation during SW revealed no statistically significant results.

**Fig. 7:**
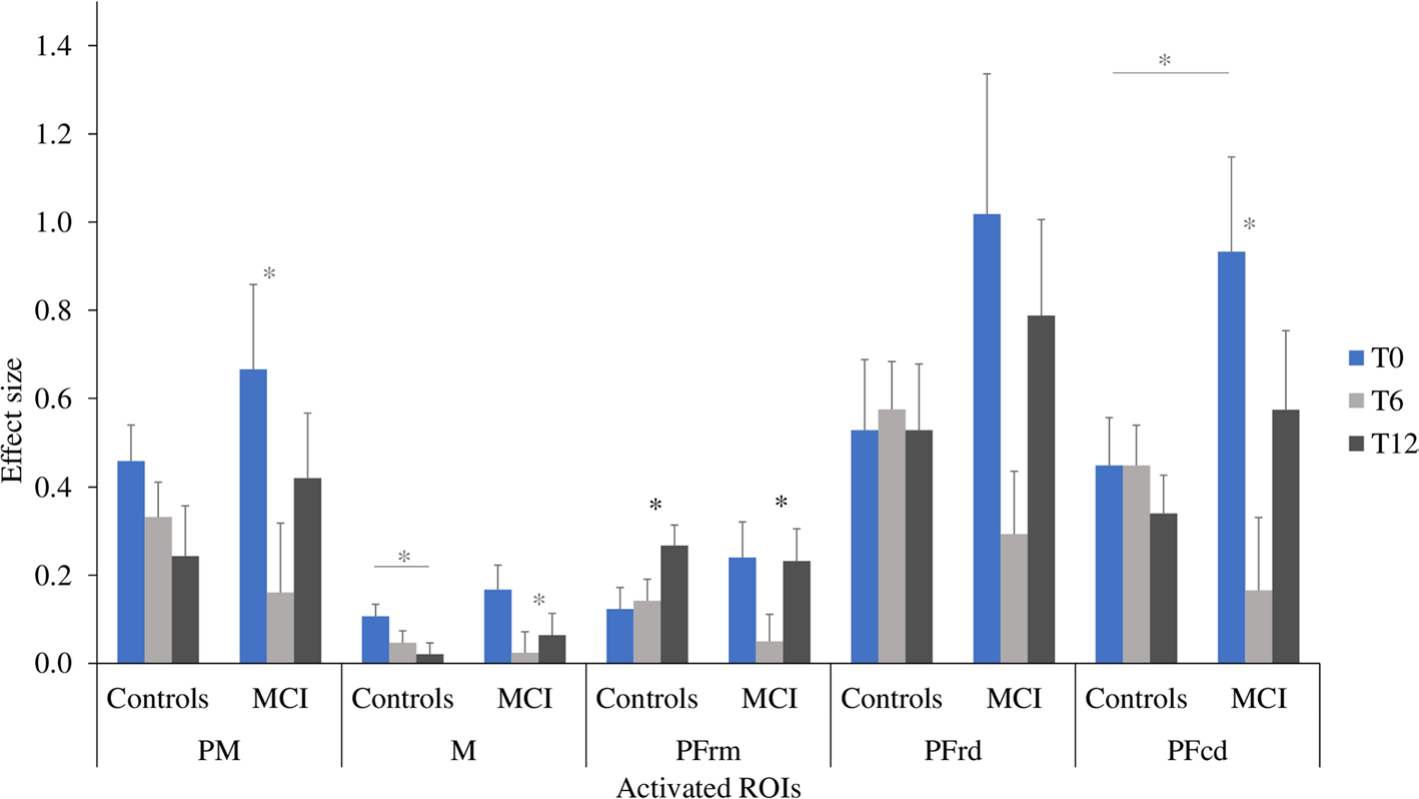
Effect sizes for HbO responses during the SC (Single Cognitive) condition in participants with MCI and healthy controls. Bars indicate standard error. * = *p* < .05

Regarding the SC condition (Fig. 7), the ANCOVAs showed a main effect of time in PFrm [F(2, 56) = 3.818, *p* = .028, η^2^_p_ = .120], where the evoked HbO response was greater at T12 than at T6 (*p* = .005). No group differences were observed. Time by group interactions tended to significance in the following regions: M [F(2, 56) = 3.086, *p* = .054, η^2^_p_ = .099], in which healthy controls showed lower HbO response at T12 than at T0 (p = .005) and MCI showed lower HbO response at T6 compared to T12 (*p* = .015); PFcd [F(2, 56) = 2.967, *p* = .060, η^2^_p_ = .096], in which those with MCI showed lower HbO response at T6 compared to T0 (*p* = .009) and tended to show greater HbO response compared to their healthy peers at baseline (*p* = .087); PM [F(2, 56) = 2.725, *p* = .074, η^2^_p_ = .089], in which those with MCI showed lower HbO response at T6 than at T0 (*p* = .011).

**Fig. 8:**
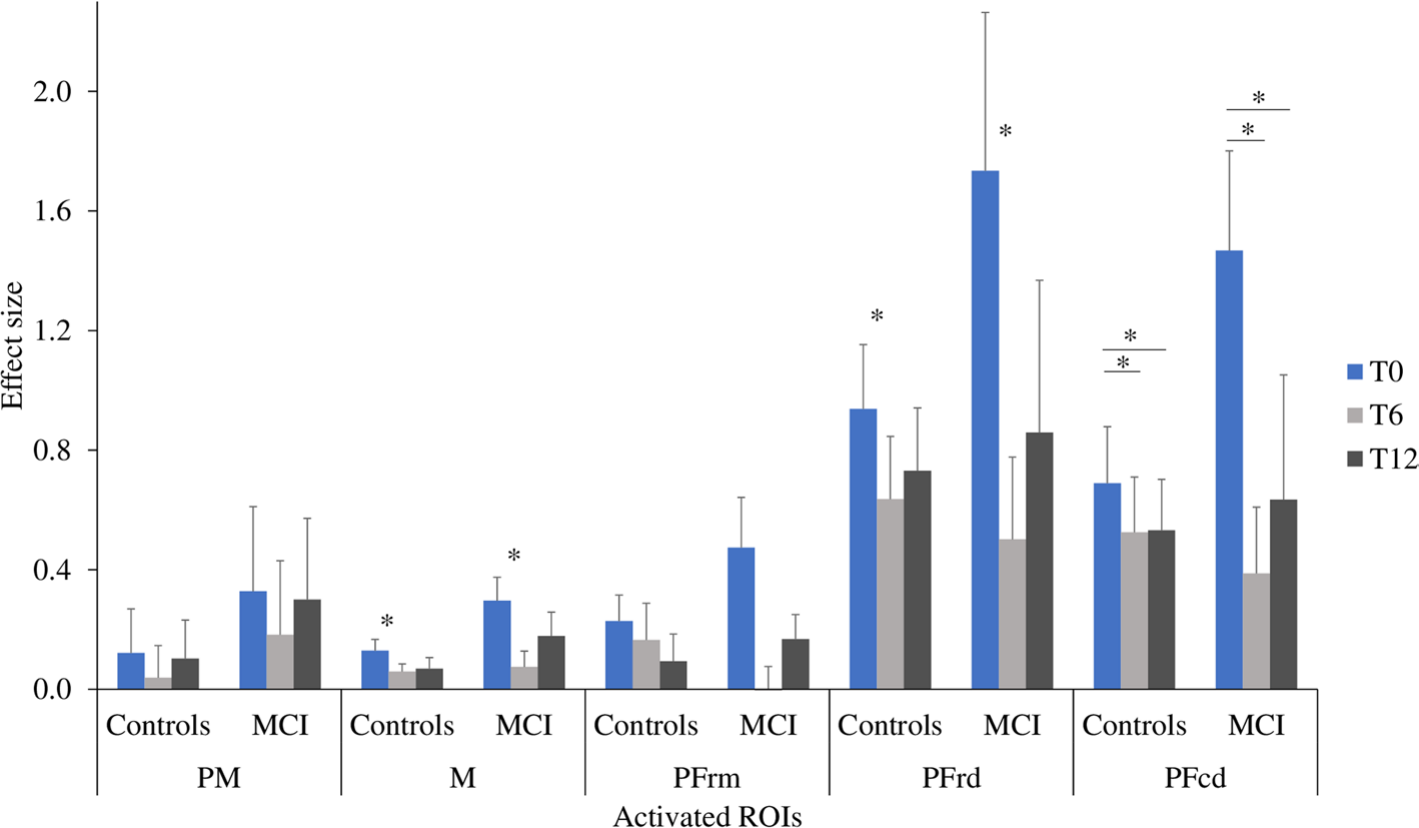
Effect sizes for HbO responses during the DT (Dual-Task) condition in participants with MCI and healthy controls. Bars indicate standard error. * = *p* < .05

Regarding the DT condition (Fig. 8), the ANCOVAs showed no group differences in the activated regions. A main effect of time was found in PFrd [F(2, 56) = 4.504, *p* = .015, η2p = .139], in M [F(2, 56) = 4.516, p = .015, η2p = .139] and in PFrm [F(2, 56) =4.360, *p* = .017, η2p = .135]. Pairwise comparisons showed that the evoked hemodynamic response was lower at T6 than at T0 (Pfrd: *p* = .051; M: *p* = .025). The pairwise comparison for PFrm showed a decreased HbO response at T6 (.036) and T12 (.107) compared to T0 (.282), but these were not statistically significant (*p* values = > .05). A main effect of time was also observed in PFcd [F(2, 56) = 5.023, *p* = .010, η2p = .152], in which the HbO response was greater at T0 than at T6 (*p* = .009) and T12 (*p* = .033). Moreover, in PFcd there was a group by time interaction that tended to significance [F(2, 56) = 3.074, *p* = .054, η2p = .099], in which those with MCI showed lower HbO response at T6 (*p* = .022) and T12 (*p* = .033) compared to T0. No other group by time interactions were found. Finally, for HbR responses there were no significant main effects of time or group and no significant time by group interactions.

Correlation analyses showed that in the MCI group there was a significant strong positive correlation in PFrm [*r* (8) = .790, *p* = .020] and a strong negative correlation in PFcd [*r* (8) = −.849, *p* = .008] between cognitive performance during the SC condition and HbO responses at T12. No other significant correlations were found in the MCI or in the control groups.

## Discussion

This pilot study explored changes in behavioral and brain oxygenation patterns in individuals with MCI and healthy controls taking part in regular PA for a period of 1 year. Behavioral results showed that cognitive performance improved at 12-month follow-up in both groups, suggesting a potentially protecting effect of PA, which was practiced regularly for the entire duration of the study, above the 150 min/week recommended by the World Health Organization [1]. Similarly, all participants showed more efficient cortical oxygenation during the 12-month period of participation in regular PA, as a decrease in task-related hemodynamic response was observed during the follow-up visits compared to baseline, with the MCI group suggesting the most benefits, at no costs to their behavioral performance.

The findings of this pilot study suggest the protective role of PA in older adulthood, and more specifically in older clinical populations. To note, the improvement in cognitive performance and more efficient brain oxygenation (i.e., reduced task-evoked hemodynamic response) were observed during both single and the dual-task conditions, thus accentuating the impact of regular PA for both low- and high-demanding cognitive tasks. Participants with MCI also walked faster at the 6-month follow-up visit than at baseline. The absence of differences in walking speed between single and dual-tasks suggests that participants, regardless of the group, prioritized the cognitive task during the DT condition, which is in line with previous literature [53]. The improvement in gait speed at T6 in the MCI group may be explained by the fact that this group reported significantly more intense PA at this specific time point compared to previous and following assessments and compared to the control group. That is, more intense PA may have accounted for greater cardiovascular and physical health, which in return caused participants with MCI to walk faster than controls. The specific improvement in gait speed and PA intensity of the MCI group at T6 also suggests that PA of greater intensity may have more beneficial effects on both cardiovascular and brain health in the long term, as shown by the reduced task-related HbO response at this time point in the MCI group. This conceptualization was also suggested by an existing investigation [54] where 6 months of biweekly resistance training contributed to better cognitive performance and brain patterns, measured by fMRI, compared to balance and tone exercises, in women with probable MCI. Given that decreased gait speed is predictive of negative outcomes in healthy older adults (e.g., cognitive decline, falls, hospitalizations) [55, 56], the increase of .07 m/s observed in our MCI group after 6 months of participation in regular PA indicates that PA may play a protective role not only on a cognitive level, but also on general physical health, in the long-term, as shown in previous investigations [57]. Our study also points out that any type of regular PA may be sufficient to show improvements in both cognition and cortical oxygenation in the MCI population. These findings are of clinical relevance because they show that any PA, when regular, may have the potential to slow cognitive and brain decline and may delay the appearance of cognitive symptoms of dementia.

fNIRS data showed task-related activation of brain regions in accordance with existing literature [58, 59]. The hemodynamic response was lower during SW, greater during SC, and increased significantly during DT in all participants. This pattern is in line with previous studies investigating cortical oxygenation during dual-task designs in both younger and older adults (for a review, see: [18]) and in individuals with MCI [32]. Specifically, bilateral rostral and dorsolateral regions of the prefrontal cortex were especially involved when the cognitive task was performed, whereas premotor and supplementary motor areas were more activated during SW. Based on the MarsAtlas cortical parcellation [52] utilized in this study, the activated regions corresponded approximately to Brodmann areas 8, 9, 10, and 46 [60], which are areas involved in the execution of complex motor and cognitive tasks and in memory processes, including working memory [61, 62]. It is thus not surprising that these areas were activated in our participants during the dual-task paradigm, a clear example of multi-tasking. An overall activation of the motor, premotor and dorsal caudal prefrontal regions was observed during the SW condition in all participants. Cortical oxygenation was similar during this task for participants in both groups and at all time points. Although the frontal cortex is associated with walking mechanisms in younger adults, it has been shown that recruitment of posterior areas is a common compensation strategy for the loss of automaticity experienced in older adulthood [63]. Therefore, it is possible that the task-related cortical activity may have been mediated by brain regions outside of the frontal regions in our participants. Future research may utilize alternative channel configurations or the use of simultaneous devices (e.g., fNIRS and electroencephalogram) to explore thoroughly the neural basis of dual-task walking in older adults.

Our primary finding of improved behavioural and cortical oxygenation over a 1-year period in MCI participants taking part in regular PA is in line with, and extends previous investigations in healthy individuals and in patients with dementia [64–66]. For instance, Ainslie et al. [65] showed better cerebrovascular health in healthy older adults who engaged in regular PA than in their sedentary healthy peers. Similarly, a recent review of MRI studies examining individuals with MCI and AD reported that general fitness had a beneficial effect on general brain functions [64]. In our pilot study we observed that individuals with and without MCI, who took part in regular PA for 1 year, tended to show lower oxygenation, but improved cognitive performance, at follow-up visits, compared to baseline measures, during both the SC and the DT components of the dual-task walking paradigm. The greater oxygenation observed at baseline, compared to other time points, is supported by the compensation-related utilization of neural circuits hypothesis (CRUNCH), stating that brain activation is greater in older adults in order to avoid poor cognitive performance related to age-related brain changes [67]. This hypothesis also proposes that, with increasing task demand, older adults reach a resource ceiling, which results in under-activation of the typically employed brain areas. Task-related cerebral under-activation is typical in individuals with MCI or AD [64, 68, 69]. However, we did not observe such a pattern in our group, who showed a decreased oxygenation and increased cognitive performance at follow-up visits. It has been previously suggested that with PA, less cortical activation is required to complete cognitive tasks in healthy individuals [70]. Our findings extended this statement to individuals with MCI, and suggest that this population may be especially sensitive to the positive effects of regular PA. Our findings also suggest that, regardless of the type, regular participation in PA may be critical to maintain a healthy brain and to prevent decline, especially in those at greater risk of cognitive decline and dementia. Finally, the correlations found between SC and HbO responses at T12 in PFrm and PFcd in the MCI group suggest that these areas may be especially sensitive to identify task-related changes in the brain and that the fNIRS may be a feasible technique to investigate this association in clinical populations. Future research may explore their role for early detection or for assessing the effect of physical interventions in the MCI population.

Some limitations of the current pilot study should be taken into account for future directions. Given the observational nature of the current study, detailed information on the type of PA in which participants engaged is not available. Moreover, PA intensity was assessed through self-reported questionnaires exclusively. Although partially limiting, the self-reported data are consistent with our behavioral and fNIRS results, which suggest changes on both cortical oxygenation and cognitive performance at follow-up visits. Future studies may consider sitting, other than standing, positions during the SC task in order to emphasize differences between single and dual tasks. The small size of our sample might also limit generalization of results, although we observed significant differences between time points (and tendencies for between-group differences) despite the small sample size, and this after controlling for sex and education, which suggests that the findings are robust and may be replicated in bigger samples. Moreover, given that the recruited participants were community dwelling and independent older adults, no information was available regarding subjective cognitive decline. Although subjective decline is a criteria to diagnose MCI in clinical settings [37, 71], subjective evidence of subtle decline is not a required criteria in the latest NIA-AA guidelines [72]. Future investigations may consider replicating our results in a controlled trial with bigger clinical samples in order to validate the casual relationship between participation in regular physical activity and changes in brain activation. Moreover, recruiting an equivalent proportion of male and female participants, would allow to better understand how biological sex can influence the pattern of cognitive performance across time in physically active males and females. Although this study included ordered numerical lists for the n-back task that differed across single and dual tasks, it would be helpful to present participants with alternative versions of this n- task at the different time points (0, 6, 12 months) in order to investigate potential dual-task walking learning effects. Although we cannot completely exclude these learning effects in the present study, the type of population, which is well-known to lack test-retest effects [73] and the nature of the experiment, which included a familiarization period at each visit, render the possibility of learning effects low. Also, a future study recruiting active as well as inactive participants could help better understand the evolution of MCI over a 1-year period according to their physical activity level. Finally, in our study we did not find time and group-related differences for HbR responses. This may have be due to a lack of statistical power for HbR values given their lower amplitude compared to HbO values [74–76]. Indeed, HbO concentration changes are believed to better reflect neurovascular coupling given that their amplitude is more sensitive to changes related to cognitive tasks [76].

## Conclusions

Although with a relatively small sample size presently, the findings of this longitudinal study suggest that participation in regular PA may improve cortical oxygenation and enhance performance on executive function tasks, the most sensitive to aging [28]. Moreover, our results provide further evidence that the fNIRS may be a powerful instrument to assess the effect of interventions in individuals with MCI. Specifically, PA should be further investigated in future research as a promising strategy to alter the trajectory of cognitive decline in seniors with MCI.

## Supplementary Information

**Additional file 1: Table S1.** Means and standard deviations of effect sizes for HbO responses during the experimental conditions.

## Data Availability

The datasets used and/or analysed during the current study available from the corresponding author on reasonable request.
