# Supplementary material for: Exploring cognitive and brain oxygenation changes over a 1-year period in physically active individuals with mild cognitive impairment: a longitudinal fNIRS pilot study
Source: BMC Geriatr. 2022 Aug 8;22:648. doi: 10.1186/s12877-022-03306-x (PMC9361664; doi:10.1186/s12877-022-03306-x)
Supplement: Supplementary file 1 — Additional file 1: Table S1. Means and standard deviations of effect sizes for HbO responses during the experimental conditions. [file 12877_2022_3306_MOESM1_ESM.docx]

Supplementary material

| **Table 1S. Means and standard deviations of effect sizes for HbO responses during the experimental conditions.** | | | | | | | | | | |
| --- | --- | --- | --- | --- | --- | --- | --- | --- | --- | --- |
| **Experimental condition** | **T0** | | | | **T6** | | **T12** | | | |
|  | **Controls** | | **MCI** | | **Controls** | **MCI** | **Controls** | | **MCI** | |
| *Single Cognitive:* |  |  | |  | |  | |  | |  |
| PM | .459 ± .346 | .666 ± .432 | | .332 ± .317 | | .161 ± .189 | | .243 ± .318 | | .420 ± .270 |
| M | .107 ± .116 | .167 ± .151 | | .047 ± .103 | | .024 ± .131 | | .021 ± .095 | | .064 ± .121 |
| PFrm | .123 ± .229 | .240 ± .212 | | .142 ± .231 | | .050 ± .166 | | .267 ± .218 | | .232 ± .219 |
| PFrd | .529 ± .720 | 1.018 ± .850 | | .575 ± .452 | | .293 ± .336 | | .529 ± .656 | | .788 ± .532 |
| PFcd | .449 ± .492 | .933 ± .421 | | .449 ± .395 | | .166 ± .401 | | .340 ± .366 | | .574 ± .395 |
| *Single Walking:* |  |  | |  | |  | |  | |  |
| PM | .116 ± .492 | .331 ± .553 | | -.021 ± .311 | | .117 ± .360 | | .158 ± .415 | | .065 ± .235 |
| Right M | .045 ± .174 | .029 ± .157 | | .008 ± .108 | | .040 ± .168 | | .026 ± .095 | | .064 ± .138 |
| Right PFcd | .072 ± .714 | .383 ± .788 | | -.223 ± .737 | | .090 ± .675 | | .003 ± .588 | | .168 ± .260 |
| *Dual Task:* |  |  | |  | |  | |  | |  |
| PM | .122 ± .495 | .328 ± .486 | | .039 ± .295 | | .182 ± .508 | | .103 ± .347 | | .300 ± .279 |
| M | .129 ± .187 | .296 ± .226 | | .059 ± .128 | | .075 ± .152 | | .069 ± .187 | | .178 ± .228 |
| PFrm | .228 ± .425 | .474 ± .477 | | .165 ± .601 | | -.046 ± .346 | | .094 ± .447 | | .168 ± .234 |
| PFrd | .938 ± 1.054 | 1.735 ± 1.497 | | .636 ±1.031 | | .502 ± .779 | | .731 ± 1.032 | | .859 ± 1.441 |
| PFcd | .689 ± .933 | 1.468 ± .943 | | .525 ± .908 | | .388 ± .626 | | .532 ± .835 | | .634 ± 1.182 |
| **Note***.* T0 = Baseline; T6 = 6-month follow-up; T12 = 12-month follow-up. | | | | | | | | | | |
